# Significance of Educational Literature and Diabetes Log Sheet on Hemoglobin A1c

**DOI:** 10.7759/cureus.21667

**Published:** 2022-01-27

**Authors:** Jaskamal Padda, Khizer Khalid, Ujala Zubair, Hussam Al Hennawi, Anwar Khedr, Vinay Patel, Ayden Charlene Cooper, Gutteridge Jean-Charles

**Affiliations:** 1 Internal Medicine, JC Medical Center, Orlando, USA; 2 Internal Medicine, AdventHealth & Orlando Health Hospital, Orlando, USA

**Keywords:** diabetes mellitus, log sheet, hba1c, microvascular complications, macrovascular complications, metabolic complications

## Abstract

Diabetes mellitus (DM) is a major cause of morbidity worldwide. The prevalence of DM has doubled over the last 35 years and is escalating. Various complications and manifestations of diabetes have caused numerous deaths worldwide, with numbers increasing every year. There have been many advances and breakthroughs over the past decade in the management of DM. The major focus of many research studies has been to evaluate effective medication combinations, preventative measures, and the way to control such morbid conditions. Our focus in this review is to discuss specific secondary prevention techniques with the diabetes log sheet and educational literature on its effectiveness in controlling diabetes. Hemoglobin A1c (HbA1c) has been accepted as a diabetes control measure in many resources worldwide. Here, we have assessed articles on the effectiveness of the diabetes log sheet and educational literature on HbA1c levels. We will begin with a few key points to acknowledge diabetes initially, followed by discussing the effectiveness of the diabetes log sheet and literature on HbA1c.

## Introduction and background

Diabetes mellitus (DM) is considered to be a growing epidemic [1]. DM is a group of metabolic diseases marked by hyperglycemia, which is caused by changes in insulin production, insulin action, or a combination of both. The persistent hyperglycemia in diabetes has been linked to long-term damage, dysfunction, and failure of various organs, particularly the eyes, kidneys, nerves, heart, and blood vessels [2]. DM is classified into four main categories: type 1 diabetes mellitus (T1DM), type 2 diabetes mellitus (T2DM), gestational DM, and other specific types of diabetes. Specific types of diabetes can be drug/chemical-induced, caused by genetics, and exocrine pancreatic disorders [3]. According to the International Diabetes Federation 2021 data, approximately 537 million individuals were diagnosed with DM, and it is estimated to increase to 643 million by 2030. The mortality rate was estimated at 6.7 million deaths due to DM in 2021 [4,5]. Additionally, DM has caused a significant economic strain on society. In 2017, the overall projected cost of diabetes within the United States was $327 billion. Direct medical expenses accounted for $237 billion, while $90 billion were attributed to decreased productivity during work, DM-related unemployment, and disabilities. Moreover, premature deaths due to DM account for a part of this economic burden [6]. The American Diabetes Association (ADA) has declared hemoglobin A1c (HbA1c) as the gold standard test to diagnose DM, requiring a threshold of 6.5%. HbA1c represents the average blood glucose levels over a three-month period. Moreover, it correlates with microvascular and macrovascular complications of DM. HbA1c also plays a vital role in managing DM and is widely utilized to measure the adequacy of glycemic control [7]. Various factors influence glycemic control such as genetics, physiology, and the quality of medical care. However, self-management strategies such as blood glucose monitoring are crucial to obtaining proper glycemic control [8]. Implementing diabetes educational programs has been shown to be associated with better outcomes [9]. In this review, we discuss the importance of educational literature and the diabetes log sheet on HbA1c levels along with glycemic control in patients with DM.

## Review

What is diabetes?

DM is a condition mainly characterized by high blood glucose levels, which increase the risk of microvascular damage such as retinopathy, nephropathy, and neuropathy. It also leads to an increased risk of macrovascular complications such as ischemic heart disease, stroke, and peripheral vascular disease. It is associated with shorter life expectancy, increased morbidity from diabetes-related microvascular and macrovascular complications, as well as poor quality of life (QOL) [7,10]. DM is classified into four main types (Table 1) [2,11].

**Table 1:** Classification of diabetes mellitus. DM: diabetes mellitus; T2DM: type 2 diabetes mellitus

DM classification	Causes
Type 1	Caused by absolute insulin deficiency resulting from B-cell destruction
Type 2	Caused by relative defects of insulin secretion, insulin resistance, or both
Gestational	DM diagnosed during pregnancy, usually due to unrecognized glucose intolerance or T2DM
Others (monogenic)	Caused by genetic defects in B-cell function, genetic defects in insulin action, diseases of the exocrine pancreas, such as cystic fibrosis, endocrinopathies, such as acromegaly, and pancreatic dysfunction induced by drugs, chemicals, or infections

What is HbA1c?

HbA1c, also referred to as glycosylated hemoglobin, is used to describe a set of stable minor hemoglobin components produced by gradual non-enzymatic reactions between hemoglobin and glucose. The rate of formation of HbA1c is directly proportional to blood glucose levels. Erythrocytes are freely permeable to glucose molecules. Inside the erythrocytes, stable bonds form between glucose and hemoglobin, which persist for 120 days, the lifespan of erythrocytes. Therefore, the level of HbA1C in a blood sample reflects the glycemic history of the preceding three months. As a result, HbA1c is the most accurate test for determining glycemic control in the previous two to three months. However, recent blood glucose levels in the last 30 days affect HbA1c more than older levels, accounting for nearly 50% of the value of HbA1c. On the contrary, blood glucose levels more than 90 days before the test contribute approximately to 10% of the result [12]. The ADA recommends using HbA1C to assess the glycemic status at least twice a year in diabetic patients meeting treatment goals and at least every three months in patients not meeting treatment goals or those whose therapy changed [3]. Both the value and variability of HbA1c have been shown to predict most complications, all-cause mortality, and cardiovascular mortality of DM [13].

Signs and symptoms

T1DM and T2DM comprise the two most common types of diabetes [14]. In T1DM, symptoms usually include polyuria, polydipsia, polyphagia, blurry vision, weight loss, and hyperglycemia not responsive to oral agents. The presentation is different in children compared to adults; moreover, it is more acute in children, with severe symptoms of polyuria, polydipsia, and ketoacidosis. However, adults have a more gradual presentation. Even though T2DM patients are more likely to be overweight or obese, T1DM should not be ruled out in these individuals [14,15].

In T2DM, the presentation is progressive in onset with the same symptoms of marked hyperglycemia in T1DM. However, many patients with T2DM remain asymptomatic for years because hyperglycemia takes years to become severe enough to cause the characteristic symptoms of diabetes. Most patients with T2DM are obese, and even those who do not satisfy the weight criteria for obesity usually have increased abdominal fat distribution. Ketoacidosis is uncommon in T2DM and mainly arises because of other stressors such as infections [14].

Microvascular complications

Diabetic retinopathy is considered the most prevalent microvascular complication of DM. It has been estimated that it leads to almost 10,000 cases of blindness in the United States each year [16]. Diabetic retinopathy is classified into two categories, namely, background and proliferative. The duration and intensity of hyperglycemia play a significant role in the development of diabetic retinopathy. In T2DM, retinopathy may develop seven years before an established diagnosis of DM. Diabetic retinopathy is caused by several pathologic processes, including osmotic stress, oxidative stress, glycoproteins, and growth factors [16,17].

Diabetic nephropathy is another microvascular complication of DM. In the United States, it is considered the most common cause of renal failure. It affects nearly 40% of patients with T1DM and T2DM. Diabetic nephropathy is classified into two stages. The first stage is microalbuminuria or incipient nephropathy, which is characterized by the excretion of 30-299 mg/24 hours of protein. If not treated, it is followed by a second stage known as overt nephropathy, defined by excretion of more than 500 mg/24 hours of protein. The pathological processes that lead to diabetic nephropathy are similar to those of diabetic retinopathy [16,18].

Another common microvascular complication of DM is diabetic neuropathy, affecting approximately 20% of adults with DM. The ADA defines it as the occurrence of symptoms or signs of peripheral nerve dysfunction in individuals with diabetes after other causes have been ruled out. Diabetic neuropathy has similar pathologic mechanisms to diabetic retinopathy and nephropathy, and its development is also dependent on the duration and severity of hyperglycemia. The most prevalent type of neuropathy in diabetes is chronic sensorimotor distal symmetric polyneuropathy, which typically presents with tingling, numbness, burning, and electrical pain in the lower extremities [16,19].

Macrovascular complications

Macrovascular diseases such as coronary heart disease, cerebrovascular disease, and peripheral vascular disease are major complications of DM. The pathologic mechanisms are atherosclerosis, increased platelet adhesion, and hypercoagulability associated with DM. Cardiovascular disease is considered the most common cause of mortality in patients with T1DM and T2DM. Moreover, cardiovascular disease represents the most common cause of healthcare expenses among patients with DM [16].

Metabolic complications

Metabolic complications of DM occur acutely and can be life-threatening. These complications include diabetic ketoacidosis, hyperglycemic hyperosmolar syndrome, hypoglycemia, and lactic acidosis. Diabetic ketoacidosis and severe hypoglycemia are more frequent in T1DM, while hyperglycemic hyperosmolar syndrome is more prevalent in T2DM. These metabolic complications account for high morbidity and mortality in patients with DM and contribute significantly to the high cost of diabetes healthcare [20].

Diagnosis

According to the ADA, there are several ways to diagnose diabetes [21], including HbA1c, random blood glucose, oral glucose tolerance test, and fasting plasma glucose. Table 2 presents the aforementioned diagnostic tests [21-23].

**Table 2:** American Diabetes Association: diagnosing diabetes. HbA1C: hemoglobin A1C; RBG: random blood glucose; OGTT: oral glucose tolerance test; FPG: fasting plasma glucose

Test	Function
HbA1c	HbA1c measures the average blood glucose levels for an approximate three-month span. Diabetes can be diagnosed when HbA1c levels are ≥6.5%
RBG	RBG measures blood glucose levels at any random time of the day. Diabetes is associated with an RBG level of ≥200 mg/dL
OGTT	In the OGTT, the patient’s blood glucose levels are measured before and two hours after ingesting eight ounces of water with 75 g of glucose. This measures the body’s ability to process simple carbohydrates. Diagnosis can be made after two hours if blood glucose levels are ≥200 mg/dL
FPG	FPG requires a minimum of eight hours of fasting, not including water intake. Diabetes can be diagnosed in symptomatic patients with a single FPG of ≥7.0 mmol/L. In asymptomatic patients, FPG of ≥7.0 mmol/L requires a second test for confirmation

Treatment

For most patients with T2DM, metformin is started as the first-line treatment, unless it is contraindicated, such as severe renal disease, chronic heart failure, diabetic ketoacidosis, and hypersensitivity [24,25]. It is safe, affordable, and associated with a decreased risk of cardiovascular events and death. Initial therapy is based on individual factors, and the goal is usually to maintain HbA1c levels less than 7%. When HbA1c levels are greater than 1.5% of the target value, patients require dual combination therapy to achieve target HbA1c levels. Insulin is very effective and can be considered in the presence of severe hyperglycemia, particularly if complications are detected such as hypertriglyceridemia, weight loss, and ketosis [24]. Glucagon-like peptide-1 agonist and sodium-glucose cotransporter-2 inhibitors are drugs used in T2DM as glucose-lowering agents. They have also shown improvement in blood pressure control, weight loss, and cardioprotective in atherosclerotic disease [26].

In T1DM, insulin is required and is generally started at 0.4-1.0 units/kg body weight. In puberty, higher insulin doses are often needed. T1DM can be treated with multiple daily injections of basal and prandial insulin. Alternatively, continuous subcutaneous insulin infusion can be used with equal efficacy. Pancreas transplantation is reserved for patients who have very serious manifestations and complications. The role of islet transplantation is questionable and further investigation is needed [24].

Role of diabetic log sheet on HbA1c

A diabetic log sheet maintains records of the patient’s activity status, calorie intake, and dosage of drug or insulin administered. Diabetic logs have assisted providers in evaluating the management strategies for patients. With the advent of time, these measurements can be done using electronic devices which are easy to use, provide more accuracy, are widely available in the market, and are extensively recommended by physicians. Some researchers have developed specially designed electronic applications and programs to help diabetic patients in keeping a record of their blood glucose levels, along with providing them literature and motivation to help control their blood glucose [27]. There have been multiple studies illustrating the improvement in patients’ diabetic management using a diabetic log. Rossi et al. conducted a study to evaluate the role of a diabetic interactive diary among patients with T1DM. His study concluded that maintenance of a diabetic log can help in the adjustment of insulin doses and identify the degree of glucose variability in these patients along with a reduction in HbA1c levels [28]. Martos-Cabrera et al. conducted a meta-analysis regarding the use of electronic applications on HbA1c levels. These applications assist in insulin administration, diet plans, awareness programs, and motivation to control diabetes. Some applications also allowed patients to maintain a diabetic diary. The results concluded that the use of a diabetic diary can help reduce HbA1c levels compared with the control groups [29]. Paramasivam et al. concluded that HbA1c levels were significantly improved in patients with gestational diabetes, along with fewer events of hypoglycemia in patients who were assisted with the use of continuous blood glucose monitoring [30]. Hou et al. conducted a meta-analysis on mobile phone applications which helped in maintaining a diabetic log and provided educational literature for diabetes. He suggested that adjuvant use of diabetic diaries can help see positive results among patients at an early stage [31]. Poolsup et al. conducted a meta-analysis to assess the benefit of maintaining a record of blood glucose levels. They concluded that patients with HbA1c of >8% observed improvement in their HbA1c after they self-monitored their blood glucose levels [32]. HbA1c can provide an estimate of glucose levels during the past three months. It is also an independent risk factor for the future development of coronary artery disease in diabetics as well as non-diabetics, but some critical information cannot be provided by HbA1c levels. This includes glucose variability and the number of hypoglycemia and hyperglycemic events. Similarly, in some medical conditions, such as chronic kidney disease and various hemoglobinopathies, HbA1c levels are unable to provide reliable levels of blood glucose levels. In such cases, daily blood glucose measurement plays an important role in guiding further management. Several studies have concluded that patients with similar HbA1c levels can have varying levels of changes in blood glucose levels between severe hypoglycemia and hyperglycemia. Maintaining a proper diabetic log including pre-prandial and post-prandial blood glucose concentrations, activity level, calorie intake and dose of medications, or insulin administered can bring improvement in the management of diabetic patients with or without any comorbid conditions [33,34].

Education role of HbA1c

In accordance with the many challenges of managing T2DM, different studies have demonstrated varying educational resources and platforms concerning long-term diabetes management. Among various diabetic educational strategies for patients, the ADA suggests that all patients diagnosed with T2DM should receive diabetes self-management education (DSME) at the time of diagnosis [35]. DSME constitutes the most widely investigated and reported modality globally [36]. By increasing knowledge and raising awareness, DSME was found to help diabetic patients achieve desired glucose control, HbA1c levels, and optimal overall health status. DSME is a practical intervention that depends on active patient participation in self-monitoring and/or decision-making abilities [37]. The concept of DSME revolves around self-efficacy, emphasizing patients’ ability to adopt a new set of behaviors and their capabilities to change [38]. DSME delivers essential knowledge and demonstrates skills that encourage diabetic patients to perform self-care needs, lifestyle changes, along with anticipating and properly handling stress and crises. DSME includes various didactic sessions that outline educational, psychological, and behavioral alterations specific to a patient’s needs through a collaborative teaching approach. Didactic sessions vary in duration ranging from brief instructional to a more comprehensive series of sessions delivered by physicians, dieticians, and nurses [39]. Ricci-Cabello et al. concluded that DSME can lead to better glycemic control with increased diabetes knowledge and self-management behavior [40].

A clear distinction should be made between DSME and passive patient education. While patient education ensures delivering pertinent knowledge and skills to follow medical advice, DSME empowers patients’ autonomy to make decisions and enhances their problem-solving abilities in case new health challenges arise [41].

The DSME program is a holistic approach involving human factors, organizational characteristics, and interventional characteristics [42,43], whereas healthcare providers can direct interdisciplinary efforts to ensure excellent diabetes control in patients [44]. Healthcare providers encourage diabetic patients to participate in educational sessions and reinforce what is learned during subsequent sessions that may help bring about successful outcomes [45,46]. Studies have also stressed the importance of a multidisciplinary approach for providing care to diabetic patients. However, until recently, there was no clear consensus on whether DSME outcomes differ with a general practitioner, nurse, specialist, or any combination of healthcare professionals [47].

Many studies have evinced the beneficial effect of the DSME method on patients’ overall health status, QOL, health awareness, and healthcare utilization, reflecting positively on diabetes healthcare burden and treatment [48]. Moreover, several other systematic reviews and meta-analyses have described improvements in primary endpoint outcomes, including, but not limited to, better glycemic control, optimal HbA1c readings, increased weight loss, blood pressure control, and improved health behavior consciousness regarding diet and exercise [49]. In a meta-analysis conducted in 2002, the DSME group showed 0.76% and 0.24% reductions in HbA1c at immediate and four or more months of follow-up, respectively. The study also demonstrated a total of three interventions measuring QOL, with two showing substantial QOL improvements in DSME program participants [50]. Interestingly, a meta-analysis demonstrated no significant impact on HbA1c in African American participants (p = 0.08%; confidence interval (CI) = 0.40-0.23) despite QOL improvement. This further necessitates the need for a more rigorously designed DSME approach for African Americans and other ethnic minority groups [40].

Further, HbA1c represents a reliable and consistent blood glucose indicator compared to conventional fasting plasma glucose, random blood glucose, and oral glucose tolerance test in detecting T2DM. However, accessibility and affordability in implementing HbA1c testing have been an issue for resource-limited underdeveloped countries. This has made monitoring T2DM at early stages challenging. Therefore, it is practically impossible to bring and generalize evidence of effective DSME on HbA1c level in a multinational domain [51,52].

According to Bekele et al., many recently published randomized control trials have depicted successful outcomes regarding the mean difference in HbA1c as an outcome of interest. To further elaborate, Bekele et al. compared different outcomes in randomized control trials involving Asian, North American, African, and European populations with a mean intervention period of eight months and one week, with the longest spanning 24 months, while the shortest spanning one month and two weeks. Overall, patients randomly allocated to the DSME program (intervention group) experienced -0.604, (95% CI = -0.854 to -0.353) standard deviation lower in HbA1c in comparison to usual care (control group) [53]. This finding supports earlier findings on the reduction of HbA1c among DSME program-managed diabetic patients [54-56] compared to other studies which showed no significant change in HbA1c desired outcome among the intervention groups [57-59]. This was postulated by different delivery of DSME programs, intervention providers, and duration.

## Conclusions

DM is a growing epidemic with a prevalence of 537 million cases in 2021 which is expected to increase to 643 million by 2030. Overall, 6.7 million deaths were estimated to occur due to DM in 2021. DM has many complications such as blindness, diabetic nephropathy, coronary heart disease, cerebrovascular disease, and peripheral vascular disease. HbA1c reflects blood glucose levels in the past three months and is an independent marker for coronary artery disease. The use of diabetic logs and educational literature can help improve overall health status, QOL, and health awareness, leading to better glycemic control. It can improve HbA1c levels, help in maintaining optimal weight, improve activity status, and help improve their overall well-being. Access to diabetic logs and educational literature can be very easily provided to many patients online or in the form of mobile phone applications. Global implementation of the use of diabetic logs and educational literature is needed to reduce the economic burden on healthcare associated with diabetes and its complications.
